# Cancer stem cells: a metastasizing menace!

**DOI:** 10.1002/cam4.629

**Published:** 2016-01-15

**Authors:** Saurabh Bandhavkar

**Affiliations:** ^1^ Department of BiochemistryG. S. Medical College and KEM HospitalMumbaiMaharashtraIndia

**Keywords:** Cancer stem cells, drug resistance, signaling pathways, therapeutic potential

## Abstract

Cancer is one of the leading causes of death worldwide, and is estimated to be a reason of death of more than 18 billion people in the coming 5 years. Progress has been made in diagnosis and treatment of cancer; however, a sound understanding of the underlying cell biology still remains an unsolved mystery. Current treatments include a combination of radiation, surgery, and/or chemotherapy. However, these treatments are not a complete cure, aimed simply at shrinking the tumor and in majority of cases, there is a relapse of tumor. Several evidences suggest the presence of cancer stem cells (CSCs) or tumor‐initiating stem‐like cells, a small population of cells present in the tumor, capable of self‐renewal and generation of differentiated progeny. The presence of these CSCs can be attributed to the failure of cancer treatments as these cells are believed to exhibit therapy resistance. As a result, increasing attention has been given to CSC research to resolve the therapeutic problems related to cancer. Progress in this field of research has led to the development of novel strategies to treat several malignancies and has become a hot topic of discussion. In this review, we will briefly focus on the main characteristics, therapeutic implications, and perspectives of CSCs in cancer therapy.

## Introduction

The concept that cancers result from abnormal changes in normal developmental processes is very old. Research has shown that cancer cells are not all the same. Majority of cells in tumors are non‐tumorigenic and are marked by limited self‐renewal ability. Only a small subpopulation of cancer cells have the ability to self‐renew and initiate tumors. These cells are referred to as cancer stem cells (CSCs) or tumor‐initiating cells. This subset of cancer cells display two hallmark properties of stem cells; self‐renewal ability and the capacity to differentiate. Although less than 1% of the overall cancer cells have the ability to proliferate extensively and form tumors, they are the major reasons for the relapse of tumors, resistance to therapies, and metastasis [1, 2]. These CSCs may give rise to two identical daughter CSCs by undergoing a symmetrical division or they may undergo an asymmetrical division to give rise to one daughter CSC and one differentiated progenitor cell, thus increasing the number of CSCs accompanied by growth and expansion of tumor 3.

The existence of CSCs was first proposed by Dick et al. 4 in hematological malignancies. Since then several evidences have emerged in existence of CSCs. Recent studies have shown the existence of CSCs in tumors of the brain, breast, prostate, pancreas, hepatobiliary, and colorectal cancer 5. Several different theories have been postulated regarding the origin of CSCs. One theory believes that normal stem/progenitor cells give rise to CSCs by obtaining the ability to generate tumors after encountering a special genetic mutation or environmental alteration 6. These mutations may occur as a result of genomic instability or induced plasticity through oncogenes. The accumulation of such mutations can enable the cells to acquire the ability of self‐renewal and tumorigenicity. Another mechanism believed to generate CSCs, the epithelial–mesenchymal transition (EMT), is characterized by series of steps, wherein initially fibroblast‐like motile cells are formed through transformation of epithelial cells, which eventually acquire the ability to invade, migrate, and disseminate 5. Aside from being similar to stem cells with respect to self‐renewal and production of differentiated progeny, CSCs are also similar to SCs in expression of specific surface markers and in utilization of common signaling pathways. However, CSCs when transplanted into animals can form tumors, normal stem cells on the other hand, cannot. Tumorigenic activity is thus a major significant difference among the two 7. Tumors grown from tumorigenic cancer cells can be serially passaged and have shown to have a mixed population of both tumorigenic and nontumorigenic cells, thus maintaining the phenotypic heterogeneity of parent tumor 8. Aside from the cellular heterogeneity, CSCs ensure survival under genotoxic stress and therapeutic toxicity. This resistance is attributed to removal of chemotherapeutic agents by drug efflux pumps, which are involved in sequestering the drugs and hindering them from reaching transformed cells. Furthermore, CSCs have enhanced DNA damage repair machinery 5. Cumulatively, these events result in sustenance of transformed cells. Thus, CSCs properly explain the heterogeneity of tumors, its mechanism of relapse and metastasis, and also the poor outcome of current therapy. Focusing on isolating and destroying these CSCs should therefore be the ultimate goal of CSC research.

## Isolation and Identification of CSCs

Since CSCs share much in common to normal stem cells, we can exploit the properties of normal stem cells in isolating and identifying CSCs such as the presence of various cell surface markers, their ability to form spheres in nonadherent medium, and their ability to exclude certain dyes 9. Thus, several in vitro assays exist to identify CSCs such as the sphere forming assay, Hoechst dye exclusion assay, Aldefluor assay, signaling pathway identification, migration assays, and detection of surface markers. However, it should be kept in mind that these assays cannot be completely reliable in identifying CSCs, as normal stem cells may also have similar characteristics. Thus, in vivo assays are regarded as the gold standard in identifying CSCs, which includes the serial transplantation in animal models.

### Stem cell markers

The main markers used for isolation and identification of CSCs include CD133, CD24, hyaluronic acid receptor, CD44, transcription factors such as OCT‐4, SOX‐2, and drug efflux pumps such as ATP‐binding cassette (ABC) drug transporters and multidrug resistance transporter 1 (MDR1). The CD24 and CD133 markers are widely used to identify CSCs in breast and colorectal cancer 10. Magnetic Cell Sorting (MACS) is widely used technique to isolate cells based on expression of stem cell markers 9. Besides fluorescence‐activated cell sorting (FACS), flow cytometry, immunofluorescent staining, and polymerase chain reactions are also widely used to isolate and characterize CSCs 5. The concomitant study of these molecular markers of stem cells, however, is mandatory so as to characterize them completely.

### Sphere‐forming assay

When cells harvested from tumor specimens are grown in a serum‐free media supplemented with epidermal growth factor (EGF) and basic fibroblast growth factor (bFGF), CSCs as well as normal stem cells form spheres or grow into colonies. The CSC population in small‐cell lung cancer (SCLC) cell lines H446 cells was suggested by Qiu et al. They demonstrated that the in vitro clonogenic and in vivo tumorigenic potentials as well as the drug resistance had increased in H446 cell lines when they were grown in a defined serum‐free medium 11. However, a major limitation associated with this assay is that it does not detect quiescent stem cells. Further, it does not reflect the actual read out of in vivo stem cell frequency 12.

### Aldefluor assay

This assay is based on the increased activity of aldehyde dehydrogenase (ALDH) in hematopoietic, mesenchymal, endothelial, and neural stem cells. This increased activity is a hallmark of various CSCs as well. Recent studies with hematopoietic stem cells, progenitor pancreatic cells, and breast CSCs indicated the presence of various ALDH isoforms, which may be tissue and cancer specific and result in aldefluor positivity. Thus, various immunohistological studies testing the potential application of ALDH isoforms as novel cancer prognostic indicators are being carried out. Kim et al. reported that ALDH^high^ cells have high tumorigenic ability as compared to CD133^+^ and ALDH^low^ cell population 13. However, recent studies have shown that the Aldefluor‐positive population in melanoma is not that stem cell enriched as compared to Aldefluor‐negative population. Also, the stem cell population identified by this assay may be heterogeneous and may require additional markers 14.

### Hoechst Dye exclusion assay

A certain subpopulation of cancer cells, termed as the side population (SP), can efficiently efflux the fluorescent DNA binding dye, Hoechst, by an ABC transporter. These SP cells exhibit higher tumorigenecity as compared to the non‐SP cells, and thus, this assay is believed to be a method used to detect CSCs 15. The main limitation with this method is the toxicity of the dye to cells; however, this toxicity can be minimized by standardization of the concentration and incubation time. Despite its limitation, this is a preferred method for identification of CSCs as it avoids the use of diverse CSC markers 16.

### Transplantation assays

Despite its merits, in vitro assays have several limitations and therefore results of in vitro assays must be confirmed with in vivo assay. Thus, assays that emphasize on the self‐renewal and tumor propagation in vivo need to be standardized. One assay that fulfills both these criteria is the serial transplantation assay in animal models. In this assay, tumor cells are transplanted into an immunocompromised mouse and the mouse is monitored at various time points for tumor growth. The xenograft tumors must then be isolated from this mouse and transplanted into another immunocompromised mouse and this mouse should be monitored for self‐renewal and tumor initiation 5. Despite its efficiency, one disadvantage associated with this assay is that they are confounded by variables such as homing efficiency of donor cells and thus are better suited to studies of wild‐type cells and not mutant cells 17.

## MicroRNAs

MicroRNAs are around 21‐ nucleotide long noncoding RNAs that via posttranscriptional gene silencing play an important role in regulating self‐renewal, differentiation, and division of cells. Till date, more than 300 miRNAs have been identified and it is predicted that around 1000 miRNAs exist in the genome. It is estimated that approximately 30% of the protein‐coding genes of the human genome are regulated by the miRNAs, thus highlighting their importance in gene expression 18. MiRNAs have been found to regulate oncogenes, tumor suppressors, and a number of cancer‐related genes. It has been revealed that dysregulation of miRNAs expression may result in tumorigenesis. Yu et al. provided the first evidence for significant downregulation of let‐7 miRNAs in CSCs. Lin‐28, a factor that binds to let‐7 miRNA, is activated in CSCs and overexpression of this lin28 is seen in wide range of cancers [8, 19]. Further, it was found out that reduced expression of two miRNAs genes, mir‐15 and mir‐16 is often associated with chronic lymphocytic leukemia (CLL) 20. Other miRNAs implicated in tumorigenesis include, miR‐ 143 and miR‐145 which are found to be downregulated in colorectal tumors 21. Studies by Croce et al. have shown downregulation of miRNAs in breast carcinomas 22. The number of miRNAs implicated in human cancers is set to increase further with the recent availability of commercial miRNA profiling platforms. Furthermore, the number of known miRNAs is increasing, rendering all data available incomplete. Thus, identification and characterization of miRNAs may be used to facilitate patient diagnosis, prognosis, monitoring, and treatment in the oncology field.

## Signaling Pathways in Maintenance of CSCs

Since normal stem cells and CSCs share a common feature of self‐renewal, it is believed that these cells may also share the same signaling pathways. The major pathways involved in maintenance and plasticity of CSCs include the Wnt, Notch and hedgehog pathways. Besides the crosstalk between receptor tyrosine kinase (RTK) pathway and interleukin‐6 (IL‐6), Janus Kinase 1 (JAK1), signal transducer, and activator of transcription 3 (STAT‐3) signaling plays a central role in regulating CSC plasticity in solid tumors.

### Hedgehog signaling pathway

**Figure 1:**
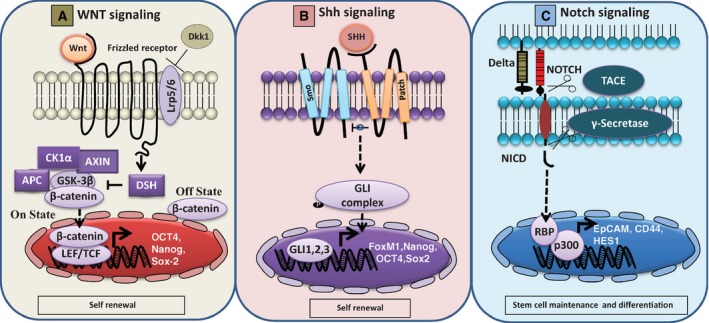
Schematic representation of Wnt, Shh, and Notch signaling cascades. Source: adapted from ref: 15.

One of the crucial pathways involved in the development and patterning during mammalian embryogenesis is the hedgehog pathway. Among the three genes such as the sonic hedgehog (Shh), Indian hedgehog (Ihh), and Desert hedgehog homolog (Dhh); the Shh shows the highest expression 23. When one of these ligands binds to the transmembrane protein receptor Patched 1 (PTCH), protein smoothened (Smo) is relieved from inhibition. This triggers the activation of GLI family of transcription factors and PTCH (Fig. 1). The aberrant activation of this pathway may contribute to tumorigenesis in many human cancers. Recent researches have suggested that the Hh pathways play a crucial role in the maintenance of various human cancers and also attributes to therapy resistance of cancer cells 5. Thus, therapeutics inhibiting any step of this pathway may prove beneficial in depletion of CSCs.

### Notch signaling pathway

This pathway plays an important role in cell–cell communication and in cell fate determination during embryogenesis and adult life. It involves ligand receptor interactions between four receptors (Notch1 – Notch4) and five ligands (Delta 1, 3, 4, and Jagged 1, 2), ultimately involved in the expression of multiple target genes 24. Upon ligand binding, the receptor is first cleaved by a metalloproteinase in the extracellular domain and subsequently in the transmembrane domain by a secretase, thereby releasing the notch intracellular domain (NICD). NICD then translocates into the nucleus and brings about activation of target genes. The Notch pathway is often found to be overactivated in a number of cancers, aiding in the maintenance of CSCs 25. It is thus believed, targeting the notch pathway may aid in eliminating CSCs 8.

### Wnt signaling

Another pathway playing an important role in embryogenesis, cell proliferation, survival, and development is the Wnt signaling pathway 26. Among the Wnt pathways, the canonical Wnt/beta‐catenin signaling is best characterized. When one of the Wnt proteins binds to a Frizzled (Fz) family receptor, the Wnt signaling begins. Lipoprotein receptor‐related protein (LRP) – 5/6, ROR 2, and RTK may act as co‐receptors to facilitate Wnt signaling. Once activated, Fz and LRP recruit the protein Disheveled (DSH). DSH further recruits glycogen synthase kinase 3 (GSK3), and LRP is phosphorylated while beta‐catenin phosphorylation is inhibited. This releases beta‐catenin from ubiquitin with which it is conjugated and is translocated to nucleus to increase the activity of target genes. Oncogenic mutations of beta‐catenin may result in dysregulation of this pathway in CSCs and thus induce neoplastic proliferation 27.

### Other signaling pathways

Aberrant mutations in activation of JAK‐STAT signaling are sufficient to induce tumorigenesis. As per recent studies, STAT3 activation is important for tumorigenic ability of CSCS in various cancers. Furthermore, a crucial role is played by IL‐6 in maintaining CSC plasticity 8. Through expression of CSC‐associated OCT2, CD44, and SOX 2 genes, regulated by IL6, non‐CSCs are converted to CSCs 24. The IL6/JAK/STAT3 pathway is important in maintaining CSC plasticity in breast cancer. Also, activation of mTORC1‐STAT3 pathway is involved in maintenance of breast CSCs 28. In non‐small‐cell lung carcinoma, the oncogenic receptor tyrosine kinases are directly involved in tumor progression and chemoresistance 29.

## Strategies Targeting CSCs

Current anticancer therapies inhibit cancer cell growth, cause cancer cells to die or a combination of both. Although initial treatments appear to be successful, a relapse generally occurs at a later date. This relapse and resistance to therapy occurs because most traditional and mainstream therapies do not target CSCs. Therefore, it is essential to target these CSCs to prevent tumor relapse and to provide an efficient and less toxic treatment for cancer therapy. Various strategies that may be employed to target CSCs include:

### Targeting the signaling pathways

Drugs such as cyclopamine and GDC‐0449 (Vismodegib) inhibit the signaling molecule smoothened (Smo) in the hedgehog pathway. These drugs are generally given in combination with arsenic trioxide (AS_2_O_3_) to increase the efficiency of the treatment 30.

Blocking the steps involved in formation of NICD is one of the most efficient methods to inhibit Notch pathway. One such class of drugs is the Gamma secretase inhibitors (GSIs), which block the Notch pathway and reduce tumor growth in vivo 31. Kondratyev et al. reported that GSI MRK‐003 inhibited the self‐renewal and proliferation of breast CSCs and was thus successful in eliminating CSCs 32.

The Wnt signaling can be inhibited by Wnt antagonists and by conditional knockout of beta‐catenin. Nonsteroidal anti‐inflammatory drugs (NSAID) inhibit the Wnt pathway by targeting the enzyme cyclooxygenase 2(aspirin) or by promoting degradation of T‐Cell Factors (celecoxib) 5.

Rapamycin inhibits the mTOR pathway by targeting MTORC1 in malignant gliomas, breast cancer, and pancreatic cancer 28. Ursolic acid acts on the STAT3 pathway to downregulate CSC proliferation in colon cancer cells 33.

### Targeting the CSC markers

Metformin is one of the most significant drugs that decrease CSC population. This can be seen by the decreased expression of CSC markers such as CD133, CD44, CXCR4, and SSEA‐1 34. Another important drug is salinomycin, which targets CD133^+^ CSCs. Pancreatic cancer in xenograft mice has been eradicated by salinomycin and gemcitabine 35.

A novel drug named cabozantinib has been identified to inhibit c‐MET, a CSC marker 36.

Use of diethylaminobenzaldehyde (DEAB) or all‐trans retinoic acid (ATRA) has sensitized ALDH^hi^CD44^+^ cells to chemotherapy or radiotherapy 37.

The combined use of inhibitors which specifically target ABC transporters of CSCs will offer a powerful strategy to eradicate CSCs 38.

### Targeting CSCs by manipulation of miRNA expression

A powerful technique for therapeutic targeting of miRNAs is antisense oligonucleotide (ASO) inhibition. Nozawa et al. showed that siRNA could downregulate EGFR and inhibit head and neck squamous cell carcinoma (HNSCC) and they also showed that this increased the sensitivity of HNSCC to cisplatin, 5‐FU, and docetaxel. Thus, microRNA‐based therapeutics are of great potential in cancer therapy 39.

### Targeting CSCs by induction of apoptosis and CSC differentiation

Apoptosis can be induced in CSCs by p53‐based drug therapy or by targeting survivin.

p53 protein in its wild type is responsible for tumor suppression; however, mutation in p53 leads to a gain of oncogenic function. Such mutated p53 can be targeted by several drugs so as to restore the wild‐type p53. One example is Phikan083 40.

Survivin on the other hand is an inhibitor of apoptosis protein which is overexpressed in various cancers, as a result of which the cancer cells are resistant to apoptosis. Targeting survivin for cancer intervention is made possible through the use of ASOs, which sensitize these cells to chemotherapy 41.

Histone deacetylase (HDAC) inhibitors, suberoylanilide hydroxamic acid (SAHA) can cause growth arrest, differentiation, and/or apoptosis of many tumor types in vivo and in vitro and has been extensively used in cancer differentiation therapy 42.

## Conclusion

Cancer stem cells are responsible not only for initiation, development, and metastasis of tumor, but they also attribute to therapeutic resistance. Current treatments target majorly the ‐CSCs and thus fail to completely cure cancer. Although various methods are available to isolate and identify CSCs, there are various limitations associated with each method and therefore there is a need to identify improved methods for isolating CSCs. In order to control the aggressiveness of cancer, novel therapeutic agents should target CSCs and important molecules in the signaling pathways. Such combination may likely yield dramatic results. Furthermore, since CSCs share many properties with normal stem cells, targeting CSCs may affect normal stem cells as well. Thus, high‐precision therapies selectively targeting CSCs while sparing normal stem cells need to be devised.

## Conflict of Interest

None declared.
